# MIRA-1, a p53^mut^ reactivator, is active on Temozolomide-resistant glioblastoma in vitro

**DOI:** 10.1186/s43556-025-00335-x

**Published:** 2025-11-26

**Authors:** Juan Perdomo, Catherine Gratas, François Paris, Francois M. Vallette, Lisa Oliver

**Affiliations:** 1 Nantes Université, INSERM, CRCI2NA - INSERM U1307, Nantes, 4407 France; 2 Departamento de Bioquímica y Biología Molecular, Instituto Universitario de Investigaciones Biomédicas y Sanitarias, Universidad de Las Palmas de Gran Canaria, Las Palmas de Gran Canaria, 35016 Spain; 3 Centre Hospitalier-Universitaire (CHU) de Nantes, Nantes, 44007 France; 4 Institut de Cancérologie de l’Ouest, Saint-Herblain, 44805 France

Dear Editor,

Glioblastoma (GBM), the main groups of primary brain cancers in adults, are highly heterogeneous tumors, a feature likely responsible for the resistance to treatments [1]. No substantial progress has been made in the treatment of this deadly tumor over the past 20 years, and the current treatment is still based on surgery (when possible) followed by combined radiotherapy and chemotherapy; mainly with the DNA alkylating agent Temozolomide (TMZ) [1]. However, the use of TMZ is efficient only in GBM that do not express the DNA repair protein, O^6^-methylguanine-DNA methyltransferase (MGMT). Unfortunately, primary GBM, after treated, very frequently relapse usually as MGMT-expressing secondary tumors and the mechanisms behind recurrence are not completely understood. The need to understand, prevent or combat recurrence remains a major goal in the treatment of this uncurable cancer [1]. One of the main factors implicated in the control of cell fate is the protein p53, which is frequently mutated in many cancers [2]. Indeed, p53 expression is altered in nearly half of GBM although these mutations appear to have no direct effect on patient outcome [2]. This is probably why very few publications have studied the impact of restoring p53 activity in GBM with mutated p53, although it is a potentially important target in other cancers [2]. We wish to address this question in the present work.

We have analyzed the response of GBM cell-lines to the small molecule MIRA-1 (mut-p53-dependent induction of rapid apoptosis), a reactivator of mutated p53 (p53^mut^) [3]. MIRA-1 has been reported to induce apoptosis in p53^mut^ cancers but also to inhibit Werner syndrome helicase (WRN) activity and thus, to interfere with the DNA repair activity [3, 4]. As shown in Fig. 1a, MIRA-1 decreased cell number in a dose-dependent manner in the p53^mut^ U251 GBM cell-line, which does not express MGMT, while a marked increase in the sensitivity to MIRA-1 was observed in a U251 TMZ-resistant cell-line (U251-R). The latter cell-line, which expresses MGMT and possesses a predominant mesenchymal signature, has been previously established by our group [5]. Figure 1a showed that the p53^wt^ U87 GBM cell-line displays a sensitivity to MIRA-1 similar to that of U251-R. U87 cells which express a low level of MGMT, are nonetheless resistant to TMZ [5]. Our results suggest that mechanisms involving p53 are at work in the resistance to TMZ but with no correlation with MGMT expression.

**Fig. 1:**
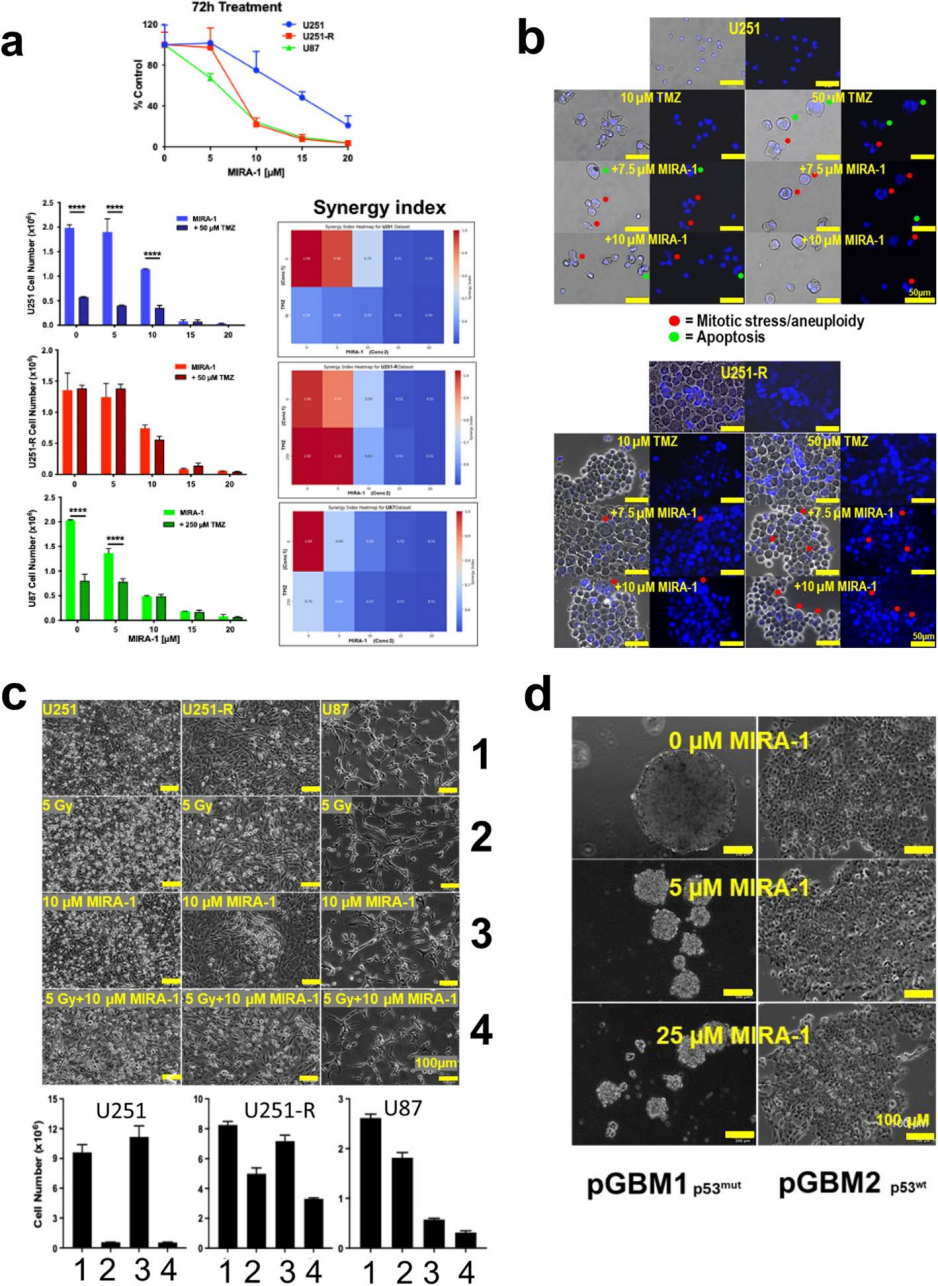
**a** Left: Dose-dependent effect of MIRA-1 on the proliferation of U251, U251-R and U87 cells. 10^6^ cells were treated with increasing concentrations of MIRA-1 (0, 5, 10, 15, 20 µM) for 3 days. Right: Heatmap visualization of synergy index values between TMZ (drug 1) and MIRA-1 (drug 2) for U251, U251-R and U87. Data shown are representative of 3 cultures. ** *p* > 0.005; **** *p* > 0.0001. **b** Hoechst staining of nuclei of U251 and U251-R cells after treatment with TMZ (0, 10 or 50 µM) and MIRA-1 (0, 7,5 or 10 µM) for 10 days. Data shown are representative of 3 cultures. Scale bar = 50 µm. **c** Effect of 5 Gy on U251 or U251-R ± 10 µM MIRA-1 for 72 h grown as described in Fig. 1a. 1 = control; 2 = Irradiation (5 Gy); 3 = MIRA-1; 4 = Irradiation + MIRA-1. Data shown are representative of 3 cultures. Scale bar = 100 µm. **d** Primary cultures derived from patient GBM tumors were obtained under 3D conditions as described in supplementary information section. pGBM1 is a p53^mut^ tumor and pGBM2 is a p53^wt^ tumor. Scale bar = 100 µm. Data shown are representative of 3 cultures

Next, we examined the effect of a combination of TMZ plus MIRA-1 on these GBM cell-lines. As shown in Fig. 1a, little or no synergy was observed between the two molecules in the different cell lines at different MIRA-1 concentrations plus 50 mM TMZ for U251 or 250 mM for U87, the effective TMZ doses for inducing cell death in these cell lines [5].

We further examined the effect of a combined MIRA-1 plus TMZ treatment on the cell and nuclei morphologies of U251 and U251-R cells to identify the type of cell death (illustrated in Fig. 1b). We found that MIRA-1 enhanced apoptosis in TMZ-treated U251-R (nuclei condensation) while TMZ alone had no effect on the induction of U251-R cell death as previously reported [5]. Of note, MIRA-1 induced apoptosis in both cell lines while mitotic catastrophe (i.e. polyploidy and aneuploidy) was detected only in U251 cells at the higher concentrations of TMZ as shown in Fig. 1b. Interestingly, U87 cells behaved like U251 cells under similar conditions (data not shown). Thus MIRA-1 appeared to induce apoptosis only in U251-R while both mitotic catastrophe and apoptosis were observed in U251 and U87 cells.

Since radiotherapy is part of the therapeutic regimen for GBM [1], GBM cells were treated with MIRA-1 plus radiation. The results obtained show that MIRA-1 increases the efficiency in vitro of radiation of 5 Gy in U251-R cells but not in U251 cells (Fig. 1c). It should be noted that, in this study, we used a TMZ concentration (50 µM) and radiation regimen (5 Gy) similar to therapeutic doses used in patients. These results suggest that MIRA-1 and TMZ act separately through distinct mechanisms, a feature that could be useful in GBM known for being composed of heterogeneous cell populations [1].

Next, we used 3D primary cultures derived from GBM patients to analyze the effect of MIRA-1 in an in vitro system. Indeed, recent findings indicate that 3D cultures respond more effectively to p53 modulation than cell lines [2]. As shown in Fig. 1d, the p53^mut^ GBM culture proliferation was more affected by MIRA-1 treatment than the p53^wt^ GBM culture.

Altogether, in this study, we have established that p53^mut^ U251 cells response to MIRA-1 is improved when resistance to TMZ is acquired and/or in irradiated cells. We found that the efficacy of MIRA-1 on TMZ-resistant cells was not related to the expression of MGMT since both U87 (a MGMT-deficient cell line) and U251^MGMT^ cells responded as shown in Fig. 1a. We observed that MIRA-1 increased cell death by apoptosis in U251 and U251-R (shown in Fig. 1b-c) but did not affect the cell cycle (data not shown). Our results agree with several publications underlying the role of apoptosis and DNA repair in MIRA-1-induced cell death. Of course, several other mechanisms and/or pathways related to p53 could be affected such as metabolism, migration and non-apoptotic cell death. More importantly, we also found that GBM primary cultures were affected by MIRA-1 especially when harboring p53^mut^ as shown in Fig. 1d. Our findings require more work, both in vitro and in preclinical models to establish its relevance and the possible undesirable side effects of p53 modulations [2].

In conclusion, our study provides a novel approach to treat TMZ-resistant GBM by targeting p53^mut^ cell lines and primary cultures. This is among the first studies to show that targeting p53 efficiently affects both conventional chemo- and radio- therapies. GBM is a deadly cancer mainly because of the lack of proper therapeutic tools for recurrent tumours. Our work suggests that targeting p53 with MIRA-1 is a putative new strategy to treat recurrent GBM. This approach represents potentially a breakthrough in the clinical handling of this deadly cancer.

## Supplementary Information

Supplementary Material 1.

## Data Availability

Data and materials are available upon request to the corresponding author (francois.vallette@univ-nantes.fr).

## Abbreviations

GBM: Glioblastoma
MGMT: O^6^-methylguanine-DNA methyl-transferase
MIRA-1: Mut-p52-dependent induction of rapid apoptosis
p53^mut^: Mutated p53
TMZ: Temozolomide
WRN: Werner syndrome helicase
