# Supplementary material for: MIRA-1, a p53mut reactivator, is active on Temozolomide-resistant glioblastoma in vitro
Source: Mol Biomed. 2025 Nov 26;6:121. doi: 10.1186/s43556-025-00335-x (PMC12657687; doi:10.1186/s43556-025-00335-x)
Supplement: Supplementary file 1 — Supplementary Material 1. [file 43556_2025_335_MOESM1_ESM.pdf]

Supplementary information

MIRA-1, a $p53^{mut}$ reactivator, is active on Temozolomide resistant Glioblastoma *in vitro* by Juan PERDOMO, Catherine GRATAS, François PARIS, Francois M. VALLETTE and Lisa OLIVER

Materials

Temozolomide (FT-GGT872) was purchased from Interchim (Montluçon, France), and all other drugs were purchased from Sigma (Saint Louis, MO). All cell culture products were obtained from Life Technologies (Carlsbad, CA).

Methods

Synergy index for TMZ and MIRA-1 in the U251, U251-R and U87 datasets have been calculated using the Bliss Independence model using Python. The results include the observed response, expected response, and the synergy index for each concentration combination.

Statistics: Data were analysed from at least 3 independent experiments and statistical analyses were performed using GraphPad Prism 9.00 (GraphPad Software, San Diego, CA, USA).

Cell lines: U251, U251-R and U87 GBM cell lines were obtained and grown as previously described [3]. Briefly, GBM cell lines were cultured in DMEM (4.5 g/L glucose) enriched with 10% FCS. U87 cells were cultured in DMEM (1 g/L glucose) supplemented with 10% FCS. All media contained 100 U/ml penicillin, 100 µg/mL streptomycin and 2 mM L-glutamine. Cells were maintained in 5% CO_2 at 37 °C. U251 cell line authentication was certified by Eurofins Genomics (Ebersberg, Germany). All cell lines were routinely tested mycoplasma-free as described in Rabé et al. [1]

Cell Counts for cytotoxicity and proliferation assays: MTT and Hoechst assays were performed as previously described [1] and in https://biotium.com/tech-tips/protocol-staining-cells-with-hoechst-or-dapi-nuclear-stains. Viable cell counts were performed using the Countess optics and image automated cell counter (Invitrogen, CA), after staining. Data are presented as the percentage of viable cells after treatment compared to untreated cells.

Primary cultures: Tumor samples classified as Glioblastoma (GBM), based on the World Health Organization criteria were obtained after informed consent from patients undergoing surgical intervention at the Department of Neurosurgery at "Centre Hospitalier Universitaire de Nantes" and the "Tumorothèque IRCNA". Within 4 h after surgical removal, Patient-Derived Cells (PDCs) were recuperated after mechanical dissociation as described earlier [2,3]. All procedures involving human participants were in accordance with the ethical standards of the ethic national research committee and with

the 1964 Helsinki declaration and its later amendments or comparable ethical standards. Primary GBM cells were cultured in defined medium (DMEM/F12 supplemented with 2 mM L-glutamine, N2 and B27 supplement, 2 µg/ml heparin, 20 ng/ml EGF, 40 ng/ml bFGF, 100 U/ml penicillin and 100 µg/ml streptomycin). All the experiments with primary GBM cells were performed at early passages. Cells were analyzed for mycoplasma regularly. The primary cultures were used for a limited number of passages (5 to 7) to avoid cellular derivation.

References

1. Rabé M, Dumont S, Álvarez-Arenas A, Janati H, Belmonte-Beitia J, Calvo GF, Thibault-Carpentier C, Séry Q, Chauvin C, Joalland N, Briand F, Blandin S, Scotet E, Pecqueur C, Clairambault J, Oliver L, Perez-Garcia V, Nadaradjane A, Cartron PF, Gratas C, Vallette FM. Identification of a transient state during the acquisition of temozolomide resistance in glioblastoma. Cell Death Dis. 2020 Jan 6;11(1):19. doi: 10.1038/s41419-019-2200-2. PMID: 31907355; PMCID: PMC6944699.
2. Oliver L, Álvarez-Arenas A, Salaud C, Jiménez-Sanchez J, Calvo GF, Belmonte-Beitia J, Blandin S, Vidal L, Pérez V, Heymann D, Vallette FM. A Simple 3D Cell Culture Method for Studying the Interactions between Human Mesenchymal Stromal/Stem Cells and Patients Derived Glioblastoma. Cancers (Basel). 2023 Feb 18;15(4):1304. doi: 10.3390/cancers15041304. PMID: 36831643; PMCID: PMC9954562.
3. Oliver L, Landais Y, Gratas C, Cartron PF, Paris F, Heymann D, Vallette FM, Serandour A. Transcriptional landscape of the interaction of human Mesenchymal Stem Cells with Glioblastoma in bioprinted co-cultures. Stem Cell Res Ther. 2024 Nov 14;15(1):424. doi: 10.1186/s13287-024-04022-6. PMID: 39538257; PMCID: PMC11562700.
